# Isolation of *Canine parvovirus* with a view to identify the prevalent serotype on the basis of partial sequence analysis

**DOI:** 10.14202/vetworld.2015.52-56

**Published:** 2015-01-13

**Authors:** Gurpreet Kaur, Mudit Chandra, P. N. Dwivedi, N. S. Sharma

**Affiliations:** Department of Veterinary Microbiology, College of Veterinary Science, Guru Angad Dev Veterinary and Animal Sciences University, Ludhiana, Punjab, India

**Keywords:** *Canine parvovirus*, madin darby canine kidney cell line, polymerase chain reaction, nested polymerase chain reaction, VP2 gene

## Abstract

**Aim:**

The aim of this study was to isolate *Canine parvovirus* (CPV) from suspected dogs on madin darby canine kidney (MDCK) cell line and its confirmation by polymerase chain reaction (PCR) and nested PCR (NPCR). Further, VP2 gene of the CPV isolates was amplified and sequenced to determine prevailing antigenic type.

**Materials and Methods:**

A total of 60 rectal swabs were collected from dogs showing signs of gastroenteritis, processed and subjected to isolation in MDCK cell line. The samples showing cytopathic effects (CPE) were confirmed by PCR and NPCR. These samples were subjected to PCR for amplification of VP2 gene of CPV, sequenced and analyzed to study the prevailing antigenic types of CPV.

**Results:**

Out of the 60 samples subjected to isolation in MDCK cell line five samples showed CPE in the form of rounding of cells, clumping of cells and finally detachment of the cells. When these samples and the two commercially available vaccines were subjected to PCR for amplification of VP2 gene, a 1710 bp product was amplified. The sequence analysis revealed that the vaccines belonged to the CPV-2 type and the samples were of CPV-2b type.

**Conclusion:**

It can be concluded from the present study that out of a total of 60 samples 5 samples exhibited CPE as observed in MDCK cell line. Sequence analysis of the VP2 gene among the samples and vaccine strains revealed that samples belonged to CPV-2b type and vaccines belonging to CPV-2.

## Introduction

Diarrhea is a very common condition in dogs caused by various viral and bacterial causative agents. It has economic implications when a large number of dogs are affected in quick succession [1]. It is multifactorial and the origin of *Canine parvovirus* (CPV) though is not absolutely clear but phylogenetically originated from feline panleukopenia virus or a very closely related carnivore parvovirus of feral canids like foxes and mink [2]. CPV was first identified in 1978 and was referred as CPV-2 after distinguishing it from CPV-1 [3].

CPV is prone to genetic evolution and has undergone several mutations that have led to various antigenic variants of CPV-2 that have replaced the original CPV-2 [4]. Currently, there are three main antigenic variants, i.e. 2a, 2b and 2c circulating in the dog population worldwide [5]. CPV-2a and CPV-2b use both canine and feline transferrin receptors for binding to cells both *in vitro* and *in vivo* [6,7] thus can infect both dogs and cats. In contrast, CPV-2 can infect both canine and feline cells *in vitro* but infects only dogs *in vivo* [2].

The genome of CPV is about 5.3 Kb and VP2 plays an important role in the determination of antigenicity and host range of CPV [8-10]. Thus, mutations affecting VP2 are mainly responsible for the evolution of different antigenic variants [4]. Thus, knowledge of genetic variations of VP2 could be of immense help in identifying emerging CPV strains in a particular geographical area, which could be used for the development of area specific appropriate vaccine strain.

The present study was undertaken to isolate CPV from suspected dogs in madin darby canine kidney (MDCK) cell line and its confirmation by polymerase chain reaction (PCR) and nested PCR (NPCR). Further, VP2 gene of the CPV isolates were amplified and sequenced to determine prevailing antigenic types.

## Materials and Methods

### Ethical approval

This study was conducted after approval by the departmental research committee and the Institutional Animal Ethics Committee

### Sample collection

Rectal swabs (n=60) were collected in phosphate buffered saline (PBS, pH 7.2) from the dogs exhibiting signs of gastroenteritis and/or hemorrhagic gastroenteritis from small animal veterinary clinics, GADVASU, Ludhiana from January, 2013 to January, 2014.

### Preparation of virus inoculum

Rectal swab in PBS was squeezed, and the tube was centrifuged at 2000 × *g* for 15 min to collect supernatant in which 400 µl of chloroform was added. It was vortexed and incubated for 10 min at 4°C and re-centrifuged at 2000 rpm for 15 min to collect the supernatant that was stored at −20°C for virus isolation.

### Isolation of virus

For the virus isolation, MDCK cell line was used in Dulbecco’s Modified Eagle’s Medium (DMEM) growth medium with 10% fetal bovine serum (FBS). In the 12- well cell culture plates when the monolayer was almost complete they were infected with 0.1 ml of the viral inoculum and incubated at 37°C for one and a half hour for the adsorption of the virus and then after washing three times, 1 ml DMEM with 1% FBS was added and incubated at 37°C for 3-4 days to observe cytopathic effects (CPE).

### Harvesting of the virus

Cell lines irrespective of the appearance of CPE was subjected to three cycles of alternative freezing and thawing and the cell culture fluid was collected in a microcentrifuge tube and stored at −20°C to be used for further passaging. The samples not exhibiting CPE in the first passage were further subjected to 2^nd^ and 3^rd^ passage following the above-indicated protocol.

### PCR and NPCR

In the CPE positive samples cell culture fluid was subjected to PCR and NPCR for confirmation of CPV. The DNA from the samples was extracted [11] and subjected to PCR by adding 15 µl of the template DNA, 5.0 µl of 10X PCR buffer (with 15 mM MgCl_2_), 1.0 µl of forward and reverse primer (25 pm/µl) each, 1.0 µl of dNTPs mix (10 mM each), 0.5 µl of MgCl_2_ (50mM), 1 U Taq DNA polymerase to make the final reaction of 50 µl using nuclease free water [12]. The reaction was put in a thermocycler (Veriti^®^, Life Technologies, USA) with 35 cycles of denaturation at 94°C for 60s, annealing at 55°C for 60 s, elongation at 72°C for 150 s and a final elongation at 72°C for 10 min. For the NPCR, primers used were as per Mizak and Rzezutka [12] following the same conditions as of PCR. NPCR reaction was set up by adding 5 µl of the PCR product, 2.5 µl of 10X PCR buffer (with 15 mM MgCl_2_), 1.0 µl each of forward and reverse primer (25 pm/µl), 1.0 µl of dNTPs (10 mM each), 0.5µl MgCl_2_ (50 mM), 1 U Taq DNA polymerase and the final volume was made up to 25 µl by adding nuclease-free water.

PCR and NPCR products (10 µl) were run using 1% agarose with ethidium bromide at 5 volts/cm with Gene Ruler ladder plus 100bp (New England Biolabs, USA). The gel was visualized and photographed using Gel documentation system (AlphaImager, USA).

### Amplification of VP2 gene

DNA from five samples exhibiting CPE in MDCK cell lines and two vaccines (Nobivac DHPPi and Megavac-6) was extracted as per Sambrook and Russell [11]. Primer pairs for the amplification of VP2 gene were designed de novo using Primer3 [13] and used in the PCR. For the amplification of VP2 gene primers were designed from the whole genome of CPV (Accession no. M19296.1) The forward primer (5’- GGTCAACCTGCTGTCAGAAA -3’) had position 2816-2835 in the genome and the reverse primer (5’- AGGTGCTAGTTGAGATTTTTCAT -3’) had position 4525-4503 in the genome. The PCR was set up by adding 1µl of the template DNA, 10µl of 5X longAmp reaction buffer (with 2 mM MgSO_4_), 1.0 µl of forward and reverse primer (25 pm/µl) each, 1.0 µl of dNTPs mix (10 mM each), 0.5µl of MgCl_2_ (50 mM), 2.5 U LongAmp Hot start Taq DNA polymerase and the reaction was made up to 50 µl using nuclease-free water. The reaction was subjected to 35 cycles at 94°C for 60 s, 58°C for 60 s, 72°C for 150 s and a finally a single elongation at 72°C for 10 min. DNA from the vaccine was considered as positive and rectal swab from a healthy dog as a negative control. PCR product was run on 1% agarose with ethidium bromide at 5 volts/cm with Gene Ruler ladder 1Kb (New England Biolabs, USA) and was visualized and photographed using Gel documentation system (AlphaImager, USA).

### Sequence analysis

For the sequencing PCR products were purified using Ultra Clean PCR cleanUp kit (MoBio Labs., Inc., USA) and the cleaned PCR products was sequenced from University of Delhi South Campus (UDSC), New Delhi. It was analyzed and compared with the available CPV sequences in the gene bank using NCBI BLAST http://blast.ncbi.nlm.nih.gov/blast.cgi [14] and Clustal Omega www.ebi.ac.uk/tools/msa/clustalo/[15].

## Results

**Figure-1:**
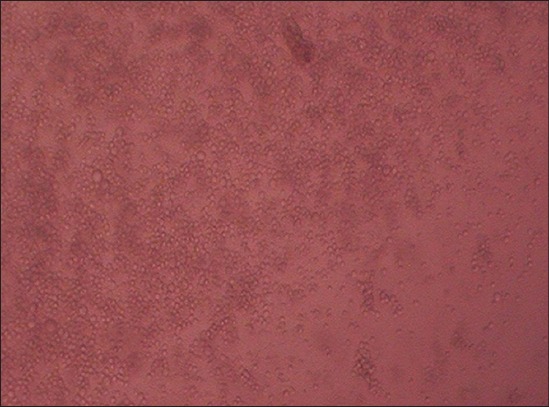
Detachment of cells 72 h post-infection of madin darby canine kidney cell line at 10X magnification

A total of 60 rectal swabs collected from January, 13-January 14 were processed for isolation in MDCK cell line when the monolayer was 80-90% complete. Out of 60 samples five exhibited CPE in the form of rounding within 24 h, clumping within 48 h and detachment within 72 h (Figure-1) of incubation.

Out of the five, four samples (P14, P41, P44, and P45) showed CPE in the first passage whereas P89 showed CPE in the 2^nd^ passage. All the remaining samples did not exhibit any CPE even after the 3^rd^ passage and were indicated negative.

Casewise analysis revealed that three CPE positive samples were from the dogs having hemorrhagic gastroenteritis; three CPE positive samples were from vaccinated dogs and three CPE positive samples were in the age group of 1-3 months (Table-1).

**Table-1:** Description of samples exhibiting cytopathic effects in MDCK cell line

Sample	Age (months)	Sex	Breed	Gastroenteritis	Vaccination status	Type of vaccine	Booster vaccination
P14	2.5	Male	German shepherd	Non-hemorrhagic	Done	DHPPi	No
P41	5	Female	Saint bernard	Non-hemorrhagic	Not done	-	-
P44	6	Female	Labredor	Hemorrhagic	Done	DHPPi	Yes
P45	3	Male	Labredor	Hemorrhagic	Not done	-	-
P89	3	Male	German shepherd	Hemorrhagic	Done	DHPPi	No

MDCK=Madin darby canine kidney

The DNA extracted from the samples was subjected to PCR and NPCR confirmed CPV by amplifying a 1198 bp product in PCR and 548 bp product in NPCR.

**Figure-2:**
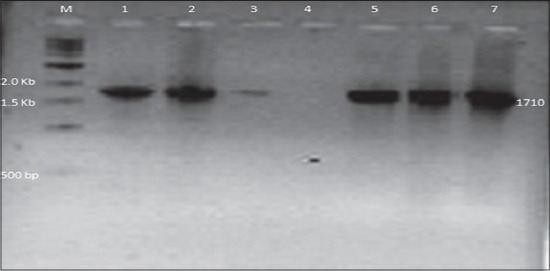
PCR for the amplification of VP2 gene of canine parvovirus. Lane M- gene ruler 1Kb, lane 1- positive control, lane 2, 3, 5, 6, 7- samples and lane 4- negative control

For the sequence analysis, complete VP2 gene was amplified from all the five positive samples using PCR. All the positive samples amplified a 1710 bp product indicative of VP2 gene (Figure-2). The PCR products were cleaned using UltraClean PCR Clean-Up kit and send to the University of Delhi South Campus (UDSC), New Delhi for sequencing and were analyzed using NCBI BLAST and multiple sequence alignment software Clustal Omega. Based on the BLAST analysis four samples (P14, P44, P45 and P89) and vaccine strains had 100% homology with CPV whereas due to some problem sample P41 could not be sequenced, so it could not be analyzed.

In the multiple sequence alignment, nucleotide sequences of the samples and the two vaccines were compared and aligned with already submitted VP2 nucleotide sequences *viz*., EU659116.1 (CPV-2), EU310373.2 (CPV-2a), JQ743893.1 (CPV-2b) and JF414822.1 (CPV-2c) revealed that there was a difference at the nucleotide position 2773, 2816, 2817, 2885, and 3189 in the samples and two vaccines with that of published sequences mentioned above depicted in the Table-2. From the multiple sequence alignment, it could be deduced that the two vaccines belonged to CPV-2 type and the samples were related to CPV-2b type.

**Table-2:** Sequence analysis of samples, vaccines and published VP2 sequences

Nucleotide position samples	2773	2816	2817	2885	3189	CPV type
EU659116.1	A	T	T	G	T	CPV-2
EU310373.2	T	C	T	A	T	CPV-2a
JQ743893.1	T	C	C	G	T	CPV-2b
JF414822.1	T	C	T	G	C	CPV-2c
V1 (DHPPi)	A	T	T	G	T	CPV-2
V2 (Megavac-6)	A	T	T	G		CPV-2
Sample P14	T	C	C	G	T	CPV-2b
Sample P44	T	C	C	G	T	CPV-2b
Sample P45	T	C	C	G	T	CPV-2b
Sample P89	T	C	C	G	-	CPV-2b

CPV=Canine parvovirus

## Discussion

CPV causes acute hemorrhagic gastroenteritis in dogs and is prone to genetic evolution mainly due to the mutations in VP2 gene [4]. It spreads rapidly in the domestic as well as in the wild population of canines. The replication of virus takes place in the villus epithelium of the small intestine that are rapidly dividing and the virus is shed in large quantity in the feces particularly 4-7 days post infection [16] and infected feces serve as a source of infection thus, rectal swabs were collected from dogs exhibiting signs of gastroenteritis both for the isolation of virus and also for the amplification of VP2 gene in the present study.

There are a number of methods that are used to diagnose CPV *viz*., virus isolation using cell culture, hemagglutination, hemagglutination inhibition, electron microscopy, indirect fluorescent test, enzyme-linked immunosorbent assay etc. [17] but PCR and NPCR are very handy and can be used for its detection as these have high sensitivity and specificity [18,19].

Rectal swabs (n=60) were collected, processed and subjected to isolation in MDCK cell line and the CPE was observed in five samples. Out of these five samples, 4 samples (P14, P41, P44 and P45) showed CPE in the first passage, but in one sample (P89) CPE was observed after the second passage. Parvoviruses are fastidious which are difficult to isolate in cell culture. Parvovirus replication is host-cell dependent and takes place only in actively dividing S-phase cells where cellular DNA polymerase is synthesized abundantly [20,21]. In an earlier study Kumar *et al*. [22] subjected 25 samples for isolation of the virus using MDCK cell line and showed that 9 samples had CPE. In another study, Nandi *et al*. [23] reported that out of a total of 13 samples 5 samples were positive by PCR but only two produced CPE in MDCK. Similarly, MohanRaj *et al*. [4] screened 77 samples by PCR and reported 51 positive using PCR but only 16 were positive when subjected to isolation in CRFK cell line.

The genome of CPV is about 5.3 Kb and VP2 gene is the immunodominant protein. VP1/VP2 gene sequence is important in the determination of antigenic types based on the epitopes located on the VP2 capsid protein. The mutations affecting VP2 are mainly responsible for the evolution of different antigenic variants [4] since this gene is under positive selection in CPV, resulting in a significantly elevated rate of molecular evolution [16]. Thus, VP2 gene was amplified, sequenced and analyzed to study the prevailing antigenic types of CPV in dogs in Punjab. Many earlier workers too have studied VP2 gene of CPV to analyze the prevailing CPV strains [23-28]. In a study, it was also reported that there is a high genetic similarity among the CPV strains, and there is substantial variation from its original strain [26].

Sequence analysis from the samples and vaccine strains revealed that all these were having 100% homology with CPV. The two vaccines were related to CPV-2 type whereas samples were related to CPV-2b type. CPV-2b has been reported as the main sub-type followed by a smaller proportion belonging to CPV-2a in India [29], which is in alignment with our study. Further, Nandi *et al*. [23] sequenced and analyzed VP1/VP2 genes of two vaccines and a field strain, revealing that the isolate belonged to CPV-2b and the vaccine strains to be of CPV-2, which is in complete agreement with the results of this study.

## Conclusion

It can be concluded from the above study that out of a total of sixty samples five samples exhibited CPE as observed in MDCK. Sequence analysis of the VP2 gene among the samples and vaccine strain revealed that samples belonged to CPV-2b type and vaccine belonging to CPV-2.

## Authors’ Contributions

GK, MC and PND designed the study. GK conducted the experiment. GK and MC analysed the data and drafted the manuscript. NSS and PND helped in providing facility to conduct the research work. All authors read and approved the final manuscript.
